# Does Noninvasive Ventilation Have a Role in Severe COPD?

**Published:** 2017

**Authors:** Nicholas S Hill

**Affiliations:** Tufts Medical Center, Boston, MA USA

Noninvasive ventilation (NIV) has long been theorized to offer benefit to patients with severe COPD. More than 30 years ago, nocturnal NIV (including negative pressure ventilators) was shown to reduce PaCO2 levels and improve respiratory muscle strength in such patients. The theories proposed that NIV would rest respiratory muscles at night, permitting improved strength and function during the daytime/ Others theorized that NIV might also improve the quality and quantity of sleep at night by preventing increases in PaCO2 and associated sleep fragmentation.

Many investigations have been performed subsequently to test these theories and until recently most showed no significant or marginal benefit. On average, it appeared that those most likely to benefit had more CO2 retention; PaCO2 > 50 mm Hg. In recent years, several studies have shown more encouraging results, although there are still conflicting findings. A retrospective study from Philadelphia showed a dramatic reduction in hospital readmissions and mortality in patients treated with nocturnal NIV after hospitalizations for acute respiratory failure. Patients in the NIV group were more obese and had more OSA than those in the non-NIV group. A randomized controlled trial from Germany using higher intensity inspiratory pressures also showed a significant reduction in mortality. More recently, the HOT HMV study from the UK presented preliminary results at the ERS meeting in London this year, showing a reduction in hospital readmissions and improved mortality. However, a Belgian trial published 2 years ago showed no benefit of NIV in patients, including no reduction in readmissions. The lecture will attempt to shed light on the controversies in this field and make recommendations on where best to use NIV for severe COPD.

